# Use of a gelatin-thrombin hemostatic matrix in obstetrics and gynecological surgery

**DOI:** 10.4274/tjod.90217

**Published:** 2018-09-03

**Authors:** Selim Mısırlıoğlu, Engin Türkgeldi, Hande Yağmur, Bülent Urman, Barış Ata

**Affiliations:** 1 Koç University Hospital, Clinic of Obstetrics and Gynecology, İstanbul, Turkey; 2 Koç University Faculty of Medicine, Department of Obstetrics and Gynecology, İstanbul, Turkey

**Keywords:** Hemostatic matrix, hemostasis, gelatin-thrombin hemostatic matrix, obstetrics, gynecological surgery

## Abstract

Gelatin-thrombin matrix (GTM) is a hemostatic sealant consisting of bovine-derived gelatin matrix and human-derived thrombin, combining both mechanical and active mechanisms to achieve hemostasis. It was approved by the Food and Drug Administration in 1999. GTM has been used by several surgical specialties; however, it is a possibly an under-used tool in obstetrics and gynecology. A limited number of studies have been performed on its use during laparoscopic endometrioma excision and myomectomy. It may prove useful in endometrioma excision in reproductive aged women because it is likely to harm ovarian reserve less than electrocautery; however, this conclusion needs to be validated. The only study on GTM use in myomectomy included 50 women randomized into GTM and control groups, and showed decreased blood loss and shorter hospital stays in the GTM group. In gynecologic oncology, it was successfully used to reduce lymphocele cases in a cohort study. GTM has been used successfully in obstetrics in a handful of cases of uncontrolled bleeding from caesarean scar, placental site, ectopic pregnancy, rectovaginal hematoma, and venous plexus over the vaginal vault after emergency postpartum hysterectomy. Risk of viral transmission is a major concern about GTM, yet there are no reports on disease transmission with GTM use to date. Rare but serious adverse effects and complications have been reported such as fatal or near-fatal thromboembolism and small bowel obstruction. Although GTM is mostly a safe product, it is still not free of complications and risks. In conclusion, although routine use of GTM cannot be recommended due to concerns about its safety, cost, and availability, it may prove useful when conventional hemostatic methods such as suturing and electrocauterization fail or are not appropriate.

## Introduction

Intraoperative hemorrhage remains a major concern of surgery in obstetrics and gynecology. Morbidity can be severe, resulting in increased transfusion rates, hospital stay, cost and rarely mortality. Although traditional methods of maintaining hemostasis (i.e., compression, suture ligation, electrocautery) usually suffice, they are not always successful or safe. In these situations, there is a need for alternative methods for achieving hemostasis. Hemostatic sealants (HS) have been developed to fulfill this need, and today a wide array of products is available. According to their mechanisms of action, they can be classified into mechanical sealants, flowable sealants, fibrin/synthetic sealants, and sealants with active ingredients^(1)^. There are more than 20 commercial products on the market, and more currently in development. A commonly used combination of bovine-derived gelatin matrix and human-derived thrombin [FloSeal Hemostatic Matrix(FloSeal) Baxter Healthcare Corporation Fremont, CA 94555, USA], has both mechanical and active ingredients to achieve hemostasis^(2,3)^. We will use the abbreviation gelatin-thrombin matrix (GTM) for, GTM in the rest of the text. GTM has been successfully used in several surgical specialties such as urology,^(4,5)^ neurosurgery,^(6,7,8,9)^ cardiovascular surgery,^(10,11)^ orthopedic surgery,^(12,13)^ and otorhinolaryngology;^(14,15,16,17)^ however, its use in obstetrics and gynecology is not similarly well-documented. This review aims to focus on the properties and use of GTM in obstetrics and gynecology.

### Properties and mechanism of action

**Figure 1:**
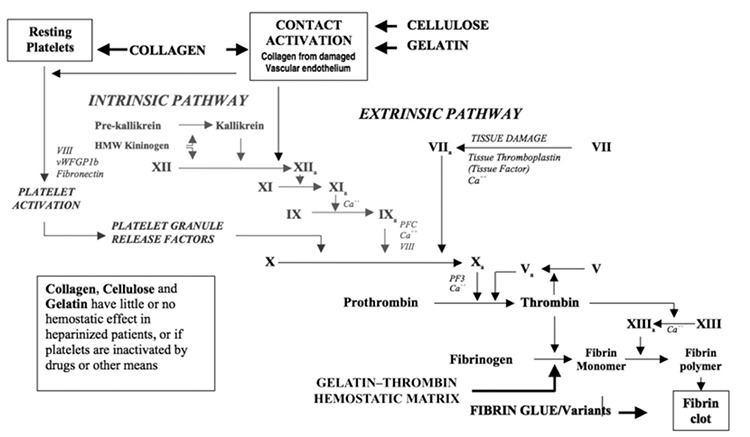
Coagulation cascade and hemostatic technologies (Courtesy of Oz et al.^(18)^ used with permission)

**Figure 2:**
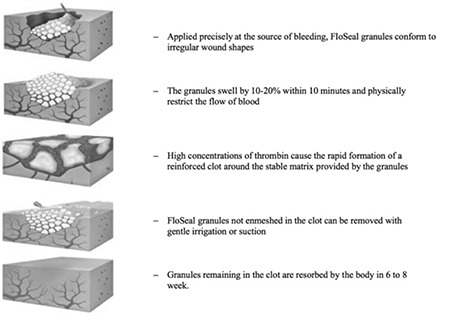
Gelatin-thrombin hemostatic matrixTM mechanism of action (Courtesy of Oz et al.^(19)^ used with permission)

The gelatin matrix is created by gelatinization of collagen extracted from bovine corium. The collagen fibers are cross-linked and stabilized with glutaraldehyde, and the thrombin is extracted from pooled human plasma. These two components are packaged separately, stored at room temperature, and mixed just prior to use^(2,18)^. GTM has two mechanisms of action (Figure 1 and Figure 2). First, the gelatin matrix fills the bleeding site with gelatin granules and swells, generating a stable clot. The gel conforms to the contour of the wound, through asymmetrical or irregular surfaces, providing a tamponade effect. A spontaneously forming clot also triggers contact activation of platelets, contributing further to hemostasis. Next, the extrinsic thrombin component of GTM converts fibrinogen into a fibrin polymer, which promotes fibrin formation at the end of the coagulation cascade. Over the course of 6-8 weeks, the GTM granules are absorbed without any residue^(18,19)^.

It is important to note that GTM functions only in the presence of fibrinogen in the clot. Therefore, it is effective in active bleeding sites exclusively. Excess product should be removed to avoid swelling. Nevertheless, care should be taken because aggressive irrigation, suction or any action that could disrupt or remove the clot itself because it would nullify the effect of GTM^(2,18,19)^. Finally, its application or injection into blood vessels should be strictly avoided because GTM intravasation may result in thromboembolism, as discussed in the “safety and adverse effects of GTM in gynecological surgery” section^(20)^. GTM was approved by the United States Food and Drug Administration (FDA) in 1999^(21)^.

### Gelatin-thrombin matrix in gynecology and obstetrics

GTM is a possibly an under-used tool in obstetrics and gynecology, as reflected in the paucity of literature regarding the related use of this substance. Studies are only available for a handful of indications, and a substantial number of publications are case reports.

### Ovarian cystectomy

Most of the studies on GTM in gynecologic surgery are in the context of ovarian cystectomies. Unlike the other investigations on GTM, their primary outcomes are the prevention of blood loss and the preservation of the ovarian reserve. Angioli et al.^(22)^ were the first to investigate the effectiveness of GTM during laparoscopic endometrioma excision. In their pilot study, they used GTM for hemostasis in the first 8 patients and bipolar forceps or carbon-dioxide laser in the following 12 patients with symptomatic endometriomas measuring ≥3 cm. Hemostasis was achieved in all patients within 3 minutes with a median time of 172 and 182 seconds in the control and GTM groups, respectively (p=0.19). Although the average blood loss was less in the GTM group, the difference was short of being statistically significant (p=0.37). Growing evidence shows a detrimental effect of endometrioma excision on ovarian reserve^(23,24,25,26,27,28)^. Alternative methods are being investigated because a possible factor is the use of electrocautery for hemostasis^(29)^. After being proven effective in endometriomas by Angioli et al.^(22)^  GTM was compared with electrocautery in three randomized controlled trials (RCT). Sönmezer et al.^(30)^ recruited 30 women with a unilateral endometriomas ≥4 cm. Despite being allocated to GTM, two patients required bipolar cautery for hemostasis and were excluded from the analysis of ovarian reserve. Preoperative and postoperative hemoglobin levels were comparable between the groups. Anti-müllerian hormone (AMH) levels were measured preoperatively, and at the first and third post-operative months. One month after surgery, the decrease in AMH was significantly higher in the bipolar cautery than the GTM group (56% vs. 29%, respectively, p=0.001); however, it was not significantly different at the third month (23% vs. 19%, respectively, p=0.467). In another RCT including 100 patients, Song et al.^(31)^ compared AMH levels between GTM and electrocautery at the third post-operative month following laparoscopic excision of endometriomas. Three patients in the GTM group required bipolar coagulation and two patients in the bipolar cautery group needed ovarian suturing for hemostasis. Estimated blood loss was similar in both groups (67.3± 49.9 mL in the bipolar cautery group vs. 55.9±45.4 mL in the GTM group, p=0.22). However, the percentage decline in AMH levels was significantly higher in the bipolar cautery group than in the GTM group (16.1% vs. 41.2%, respectively; p=0.004). The third RCT included 60 women with bilateral endometriomas^(32)^. Either GTM or bipolar coagulation was used for hemostasis, and serum AMH levels were compared between the groups at the third post-operative month. Women in the GTM group had a significantly higher mean AMH level than the bipolar cautery group (1.68±0.32 ng/dL vs. 1.08 ±0.32 ng/mL). It should be noted that this trial remained as an abstract and did not develop into a full-text article; therefore, detailed information about the methods and results are not available. In our systematic review and meta-analysis of the effect of hemostatic methods on ovarian reserve following laparoscopic endometrioma excision, we found that although bipolar cautery caused a significantly greater decline in AMH compared with alternative methods, namely GTM and suturing [95% confidence interval (CI)=-13.00, -0.90], the difference was not significant in the subgroup analysis comparing bipolar coagulation and GTM (95% CI=-14.07, 2.53)^(29)^. We concluded that although the latter was possibly a false-negative finding due to a small sample size and there was moderate quality evidence supporting its use, we were still hesitant to suggest the widespread application of GTM due to its marginal benefit, additional cost, and possible adverse effects. To the best of our knowledge, there are no published studies about the use of GTM during the excision of other types of ovarian cysts, except for a case report by Ebert et al.^(33)^ about stripping an ovarian serous cystadenoma.

### Myomectomy

Even though myomectomy is a commonly performed procedure that can cause significant blood loss, there is limited data on the use of GTM in myomectomy. Raga et al.^(34) ^randomized 50 women with symptomatic fibroids larger than the size of a 16 weeks’ pregnant uterus who were undergoing conventional myomectomy into GTM and control groups. GTM or sterile saline was applied to the fibroid bed immediately after the removal of the fibroid and before uterine wound closure. Blood loss was estimated as the sum of the weight change in the gauzes and the blood volume in the suction bottle. The average intraoperative blood loss was 80±25.5 mL (range, 25-150 mL) and 625±120.5 mL (range, 250-950 mL) for the GTM and control groups, respectively (p<0.005). Likewise, average postoperative blood loss, measured by surgical drains, was significantly more in the control group (25±5 mL vs. 250±75 mL, p<0.005). Although none of the women in the GTM group required blood transfusion, 5 patients were transfused in the control group. All of this translated into longer hospital stays for the control group (p<0.005). The small sample size and possible lack of blinding are drawbacks of this study and it is unfortunate that no other trial has been performed to support or oppose its findings.

### Gynecologic oncology

It is interesting that the use of GTM has not been reported much in gynecologic oncological surgery, a field where hemostasis is of utmost importance and bleeding from or around many vital organs or tissues is expected. Yet, its use in pelvic lymph drainage and wound healing has drawn some attention.

Han et al.^(35)^ reported the case of an 86-year-old woman who had undergone wide radical excision and bilateral inguinal lymphadenectomy for vulvar cancer. She had bilateral inguinal wound separation and excessive lymphorrhea postoperatively. Application of GTM resulted in successful granulation formation. Perfect wound healing and no drainage from the groin was reported two months later.^(35)^

In addition to this case-report, there is only one cohort article that studied the effect of GTM for the treatment pelvic lymphoceles. In this study, 50 patients underwent pelvic +/- paraaortic lymphadenectomy for various gynecologic cancers. In the study group, 5 mL GTM and its spray form (Coseal^®^) was used at the lymphadenectomy side instead of a pelvic drainage system. Pelvic drainage systems were used in the control group. Application of GTM decreased the hospital stay and the number of symptomatic lymphoceles in patients with gynecologic malignancies^(36)^.

### Ectopic pregnancy

Another scenario where a gynecologic surgeon can encounter uncontrolled bleeding is ectopic pregnancy. Although most tubal pregnancies are managed successfully with salpingectomy or salpingostomy, additional measures may be required to control bleeding. Clapp and Huang^(37)^ reported two cases of tubal ectopic pregnancies where electrocautery failed to achieve hemostasis and GTM was used successfully. Interestingly, Watrowski^(38)^ reported two cases of tubal pregnancies managed through salpingostomy where they used only GTM for hemostasis. Due to its cost, limited availability, and yet-to-be-proven efficacy, this approach is far from being the standard. It remains a viable option in patients who wish to preserve their fallopian tubes. Gorry et al.^(39)^ and Watrowski et al.^(40)^ reported cases of primary peritoneal pregnancy and primary omental pregnancy where GTM was used successfully to control bleeding.

### Obstetrics

The literature on the use of GTM in obstetrics is limited to a few cases in the context of postpartum hemorrhage. Moatti et al.^(41)^ described a case of massive post-partum hemorrhage forming a rectovaginal hematoma that could not be controlled with conventional methods; application of GTM with packing provided hemostasis. Another case, uncontrolled bleeding from the venous plexus along the vaginal vault after emergency postpartum hysterectomy in a patient with disseminated intravascular coagulopathy was managed successfully with GTM^(42)^. A patient with acute fatty liver of pregnancy who presented with acute hepatic and renal failure along with a hypofibrinogenemia was noted to have bleeding after vaginal delivery by vacuum extraction. This was controlled with intrauterine, vaginal application of GTM and recombinant activated human factor VIIa transfusion^(43)^. Finally, GTM proved successful in a woman with post-partum hemorrhage due to vaginal laceration that could not be controlled using traditional techniques due to “poor tissue quality”^(44)^. Similar cases of bleeding from a caesarean scar^(45)^ and placental site^(46,47)^ were controlled with GTM. These reports show that GTM can be an option in post-partum hemorrhage in cases where traditional methods fail.

### Safety and adverse effects of gelatin-thrombin matrix in gynecologic surgery

As with any other product that contains human or animal derived components, GTM poses a theoretical risk of viral transmission. Although this risk can be reduced by screening donors and tracing cattle, it cannot be ruled out with current technology^(6)^. There are no reports of disease transmission from the currently available GTM products in the literature. Thromboembolism is another major concern about GTM and HS in general. Fatal pulmonary thromboembolism has been reported following the use of GTM during spinal surgery in a 78-year-old woman^(20)^. She developed dyspnea with right-sided heart failure due to left pulmonary artery embolization 8 hours after surgery. Autopsy revealed that the thrombus in the pulmonary artery contained acellular eosinophilic granules with enclosed fibrin and thrombocytes, convincing the pathologists that thromboembolism was a result of embolization of GTM granules from the application site to the pulmonary artery. The authors suggested that continuous uptake of small amounts of GTM through small vessels around the paravertebral site was the cause of thromboembolism. The injury to the vessel wall might have triggered the coagulation cascade that caused the migration of GTM granules. Therefore, surgeons should be careful about the risk of intravascular thrombus formation when GTM is used around relatively large-sized vessels.

**Figure 3:**
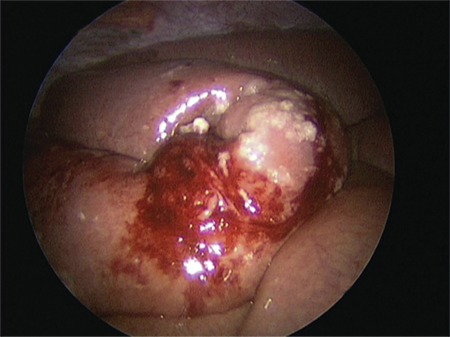
Granulation tissue and significant inflammatory reaction seen at the site of bowel adhesions (Courtesy of Suzuki et al.^(52)^ used with permission)

Another case report involving an 18-year-old woman who developed peri-operative disseminated intravascular coagulation and acute right-sided heart failure that occurred during spinal surgery immediately after the application of an absorbable gelatin powder mixed with bovine thrombin. This event was attributed to unintentional intravasation of HS^(48)^. Small bowel obstruction (SBO) is another potential serious complication after application of GTM or similar HS in the peritoneal cavity^(49,50,51,52)^. SBO following its use in gynecological surgery was first reported in 2009^(51)^. GTM was applied after iatrogenic injury to the inferior vena cava during laparoscopic lymphadenectomy for endometrial cancer. The patient developed nausea, vomiting, and abdominal pain on the 6^th^ postoperative day and bowel sounds were absent. She was initially managed conservatively, but diagnostic laparoscopy was required on the 11^th^ postoperative day. Adhesions were seen on the GTM application site only and this was thought to be the obvious cause of obstruction. A 15-cm small bowel segment was resected. Pathologic evaluation showed significant fibrotic changes caused by a foreign material, in accordance with a GTM product. Suzuki et al.^(52)^ reported two cases of laparoscopic gynecologic procedures complicated by SBO, possibly related to the use of a hemostatic agent. In the first case, a 44-year-old woman who underwent laparoscopic myomectomy, a single dose of GTM (4 mL in total) was administered to the hysterotomy site for persistent oozing. She was re-admitted with severe pelvic pain on 4^th^ postoperative day. In the second case, total laparoscopic hysterectomy and bilateral adnexectomy was performed and GTM was applied to control the bleeding from the left pelvic side wall after adhesiolysis. In both cases, SBO was noted at the GTM application sites during diagnostic laparoscopy (Figure 3). The authors concluded that triggering of an allergic reaction and formation of eosinophilic granulomatous tissue may result in intraperitoneal adhesions and SBO^(52)^. In the light of these case reports, it is suggested to wait for two minutes after GTM application and remove the excess material with gentle irrigation to decrease the risk for developing granulomatous tissue, which could result in SBO. These reports show that although GTM is mostly a safe product, it is still not free of risk. These are important considerations before its use because safer alternatives such as suturing or compression are available. GTM is a hemostat consisting of bovine-derived gelatin matrix and human-derived thrombin, combining both mechanical and active ingredient mechanisms to achieve hemostasis. Although it cannot be recommended as the first-line method due to concerns about safety, cost, and availability, it may be useful when conventional hemostatic methods such as suturing and electrocautery fail or are not appropriate. Moreover, it may prove useful in endometrioma excision in reproductive aged women because it is likely to harm ovarian reserve less than electrocautery. However, this should be validated with high quality studies. AThis research did not receive any specific grant from funding agencies in the public, commercial, or not-for-profit sectors.
